# Hyperacute total and item-level NIHSS evolution after intravenous thrombolysis in anterior-circulation ischemic stroke: a Romanian tertiary stroke center cohort

**DOI:** 10.25122/jml-2026-0127

**Published:** 2026-09

**Authors:** Irina Maria Vlad, Dafin Fior Mureșanu, Lăcrămioara Perju-Dumbravă

**Affiliations:** 1 Doctoral School, Iuliu Hațieganu University of Medicine and Pharmacy Cluj-Napoca, Romania; 2 Department of Neurosciences, Iuliu Hațieganu University of Medicine and Pharmacy, Cluj-Napoca, Romania; 3 Neurology Clinic, Cluj County Emergency Clinical Hospital, Cluj-Napoca, Romania; 4 RoNeuro Institute for Neurological Research and Diagnostic, Cluj-Napoca, Romania

**Keywords:** acute ischemic stroke, intravenous thrombolysis, National Institutes of Health Stroke Scale, early neurological improvement, hyperacute neurological recovery

## Abstract

Hyperacute neurological change after intravenous thrombolysis (IVT) is commonly summarized using the total National Institutes of Health Stroke Scale (NIHSS) score, which may obscure changes in individual deficits. This study aimed to describe total and item-level NIHSS evolution during the first 2 hours after IVT in anterior-circulation acute ischemic stroke. We retrospectively analyzed prospectively recorded data from 124 eligible IVT-treated patients at a Romanian tertiary stroke center in 2024–2025. Median age was 67 years, and 62.1% were male. The NIHSS was assessed at baseline, 1 hour, 2 hours, and discharge. Hyperacute total-score changes were analyzed descriptively and with Friedman and Wilcoxon signed-rank tests, while item-level recovery and recovered and worsened NIHSS item-points were summarized descriptively. Median NIHSS decreased from 6 (interquartile range [IQR] 5–9) at baseline to 4 (IQR 3–7) at 1 hour and 4 (IQR 2–5) at 2 hours. Improvement relative to baseline occurred in 72.6% of patients at 1 hour and 79.8% at 2 hours. Between 1 and 2 hours, 42.7% improved further, 53.2% remained unchanged, and 4.0% worsened, despite an unchanged group median. The NIHSS differed significantly across baseline, 1 hour, and 2 hours (Friedman χ^2^(2) = 152; *P* < 0.001). By 2 hours, improvement proportions were numerically higher than at 1 hour for 14 of 15 NIHSS components, and 353 item-points had been recovered among baseline-positive deficits. Hyperacute neurological evolution after IVT was heterogeneous across individual deficits. Item-level analysis characterized the neurological profile underlying aggregate change and may help contextualize subsequent monitoring, assistance, and rehabilitation needs.

## Introduction

Intravenous thrombolysis (IVT) remains an established reperfusion treatment for eligible patients with acute ischemic stroke and is recommended in contemporary European and American guidelines [1,2]. Timely treatment and systematic assessment of neurological status are central to acute stroke care. The use of a stroke severity rating scale is recommended, preferably the National Institutes of Health Stroke Scale (NIHSS) [3], to quantify neurological deficits and objectively follow changes in clinical status, with serial neurological assessments forming part of post-thrombolysis monitoring [1]. Treatment administration is followed by a period in which the patient’s neurological status may continue to change, including early neurological deterioration (END) [4,5].

The NIHSS provides a standardized measure of neurological deficit and comprises individual items assessing motor and sensory function, vision, level of consciousness (LOC), attention/neglect, language, and speech production [6,7]. The total NIHSS score summarizes these components into a single value; however, Braun *et al*. emphasized that, although the NIHSS provides an overall measure of stroke severity, the aggregate score does not indicate how individual neurological deficits contribute to recovery over time [6]. The clinical relevance of individual NIHSS components is also supported by registry data: Hagberg *et al*. found that specific admission NIHSS items were associated with functional independence at 3 months and concluded that affected NIHSS items should be considered in addition to the total score for treatment guidance and prognostication [7].

The composition of neurological recovery is clinically relevant because different stroke deficits affect patients in different ways. Post-stroke aphasia is associated with impaired functional communication and reduced quality of life, while visuospatial neglect adversely affects independence and performance in activities of daily living, including self-care and mobility, and may also restrict participation and increase caregiver burden [8,9]. The NIHSS does not, however, provide a comprehensive assessment of all these consequences, and it should not be regarded as a cognitive screening instrument. Abzhandadze *et al*. showed that an NIHSS-derived cognitive subscale had poor agreement with the Montreal Cognitive Assessment and limited sensitivity for cognitive impairment in acute stroke [10]. Similarly, NIHSS item 9 detects severe aphasia reasonably well but can miss milder language impairment when compared with dedicated language testing [11]. Thus, individual NIHSS items are best considered brief indicators of neurological functions that may warrant more detailed domain-specific evaluation.

Because stroke-related impairments differ in their functional consequences, assessment of specific deficits is also relevant to rehabilitation planning and the identification of appropriate interventions [12].

These considerations also support focusing item-level analyses on anterior-circulation stroke. The NIHSS is weighted toward deficits associated with anterior-circulation lesions and may underestimate clinical severity in posterior-circulation stroke, in which several characteristic brainstem, cerebellar, and bulbar manifestations are not adequately represented. Alemseged *et al*. showed that a posterior-circulation-specific modification of the NIHSS, incorporating additional posterior-circulation signs, had greater prognostic accuracy for 3-month outcome than the conventional NIHSS in patients with mild-to-moderate posterior-circulation stroke [13].

Very early neurological response after IVT has previously been assessed using the total NIHSS. In the SITS-EAST registry, clinical recovery was specifically evaluated at 2 and 24 hours after IVT using total NIHSS-based definitions [14]. Other contemporary studies have documented clinically important deterioration during the same hyperacute period. In patients with minor stroke and large-vessel occlusion intended for IVT alone, Seners *et al*. found that, among patients who developed END of presumed ischemic origin, 48% deteriorated within 2 hours after IVT initiation [5]. More recently, neurological deterioration occurring during thrombolytic infusion was documented in 8.4% of an IVT-treated cohort [4]. Conversely, recent item-based reperfusion research has demonstrated differential improvement across neurological functions, particularly language and motor deficits, while also examining neglect, within a large-vessel-occlusion population receiving heterogeneous treatment strategies, including IVT, endovascular thrombectomy, combined therapy, or no reperfusion treatment [15].

Previous studies have demonstrated that neurological status may change during the first hours after IVT, with both early recovery and neurological deterioration captured using serial total NIHSS assessments [4,5,14]. Other studies have highlighted the clinical information contained within individual NIHSS components and have examined recovery across specific neurological functions after reperfusion therapy [6,7,15]. Building on these complementary perspectives, the present study examines total and item-level neurological evolution together during the hyperacute post-IVT period. Because individual NIHSS items are routinely recorded during acute monitoring, serial item-level analysis can characterize both the extent of early neurological change and the specific deficits contributing to changes in the total NIHSS.

The present study aimed to describe the hyperacute neurological course during the first 2 hours after IVT in patients with anterior-circulation acute ischemic stroke. Changes in total NIHSS were assessed from admission to 1 hour and to 2 hours, including the interval between the 1- and 2-hour assessments. Item-level recovery was evaluated from admission to 1 hour and from admission to 2 hours among patients with the corresponding deficit at baseline. Recovered and worsened NIHSS item-points were also quantified to describe the magnitude and composition of early neurological change. The analysis was descriptive and focused on the extent and pattern of early neurological recovery rather than on differences in biological responsiveness or prognostic value between individual NIHSS components. The study is a retrospective analysis of prospectively recorded real-world clinical data from a Romanian tertiary stroke center.

## Material and Methods

### Study design and setting

This retrospective observational cohort study included consecutive patients treated with IVT for acute ischemic stroke involving the anterior circulation at a Romanian tertiary stroke center between 2024 and 2025.

The study was restricted to anterior-circulation ischemic stroke because the NIHSS is weighted toward deficits associated with anterior-circulation lesions and may underestimate clinical severity in posterior-circulation stroke [13].

The study was approved by the Ethics Committee of the Iuliu Hațieganu University of Medicine and Pharmacy Cluj-Napoca (approval no. 165/17 June 2026) and the Ethics Committee of Cluj County Emergency Clinical Hospital (approval no. 18622/28 May 2026) and was conducted in accordance with the Declaration of Helsinki and the applicable requirements of the General Data Protection Regulation (GDPR). Given the retrospective nature of the study, the requirement for informed consent was waived by the Ethics Committee.

### Study population

Patients were eligible if they were aged ≥18 years, had a final diagnosis of acute ischemic stroke involving the anterior circulation, received IVT, and had total NIHSS assessments available at baseline, 1 hour, 2 hours, and acute hospital discharge. Reliable thrombolysis timing information and baseline neuroimaging, including the Alberta Stroke Program Early CT Score (ASPECTS) [16] and lesion laterality, were also required.

For item-level analyses, individual NIHSS component scores were required at baseline and at the corresponding 1- and 2-hour assessments.

Patients were excluded in the presence of posterior-circulation or vertebrobasilar stroke, endovascular thrombectomy during the index stroke episode, hemorrhagic transformation during the acute hospitalization, in-hospital death, stroke mimic or a final diagnosis other than acute ischemic stroke, or transfer before an adequate discharge neurological assessment. Patients with in-hospital stroke were excluded when symptom onset, baseline NIHSS timing, or thrombolysis timing could not be reliably established. Patients with pre-existing neurological or cognitive deficits that substantially affected baseline NIHSS scoring, or with acute non-stroke conditions preventing reliable serial NIHSS assessments, were also excluded.

All patients received intravenous alteplase.

### Clinical variables

Recorded baseline demographic and clinical variables included age, sex, stroke laterality, baseline NIHSS, pre-stroke modified Rankin Scale (mRS) score [17], ASPECTS, onset-to-needle time, admission glucose, and serum creatinine. Length of hospital stay was recorded for descriptive purposes.

Recorded pre-existing comorbidities included arterial hypertension, type 2 diabetes mellitus, atrial fibrillation, previous stroke, chronic kidney disease, heart failure, coronary heart disease, peripheral artery disease, dyslipidemia, chronic gastroenterological disease, and chronic respiratory disease.

### NIHSS assessment and definition of hyperacute neurological change

Neurological deficit severity was assessed using routinely documented NIHSS examinations. Total NIHSS and individual NIHSS item scores were abstracted from clinical documentation at baseline and from predefined routine monitoring fields corresponding to 1 hour and 2 hours after initiation of IVT. NIHSS assessments were performed as part of routine clinical care by the physicians responsible for IVT administration and subsequent acute neurological monitoring. Total NIHSS at acute hospital discharge was additionally recorded as a later neurological assessment.

The baseline, 1-hour, and 2-hour examinations constituted the predefined hyperacute assessment period. Discharge NIHSS represented neurological status at a later, non-standardized clinical time point and was therefore not considered an additional fixed hyperacute interval.

Within-patient total NIHSS change was evaluated over three intervals: admission to 1 hour, admission to 2 hours, and 1 hour to 2 hours. NIHSS change was calculated as ΔNIHSS = later NIHSS − earlier NIHSS. Accordingly, negative ΔNIHSS values indicated neurological improvement, zero indicated no change, and positive values indicated worsening.

For each interval, patients were additionally classified as improved, unchanged, or worsened according to whether their total NIHSS decreased, remained identical, or increased, respectively. No proportional NIHSS reduction or data-derived responder threshold was used.

### Item-level NIHSS recovery

Item-level neurological evolution was assessed separately for each standard NIHSS component as recorded clinically.

For the admission-to-1-hour and admission-to-2-hour analyses, only patients with a non-zero score for the corresponding NIHSS item at admission were included in the recovery denominator. A lower follow-up item score was classified as improvement, an identical score as unchanged, and a higher score as worsening. The item-specific improvement rate was calculated as the number of patients with a reduction in the corresponding item score divided by the number of patients with a non-zero baseline score for that item, multiplied by 100.

As an additional descriptive analysis, item-level change from 1 to 2 hours was examined among patients who had the corresponding deficit at admission and continued to have a non-zero score for that item at 1 hour. Patients whose deficit had completely resolved by 1 hour were excluded from the second-hour recovery denominator because no further item-level improvement was possible.

### Recovered NIHSS item-points

As a complementary descriptive analysis, the magnitude of item-level neurological change was quantified using recovered and worsened NIHSS item-points. For each patient and NIHSS component, recovered points represented the magnitude of a decrease in item score, whereas worsened points represented the magnitude of an increase. These values were summed across patients for each NIHSS component. Net change was calculated as recovered points minus worsened points.

The relative contribution of each component to overall recovered NIHSS item-points was calculated as the recovered points for that component divided by the total number of recovered NIHSS item-points across all components, multiplied by 100.

The primary item-point analyses were performed from admission to 1 hour and from admission to 2 hours. The 1-to-2-hour item-point analysis was performed as an additional descriptive analysis and is reported in the Supplementary Material. The recovered-point contribution was interpreted separately from the item-specific improvement rate, as it reflects the numerical contribution of an NIHSS component to total score recovery rather than the proportion of patients with that deficit who improved.

### Study outcomes

The main outcomes of interest were total and item-level NIHSS evolution during the first 2 hours after IVT. Total NIHSS change was assessed from admission to 1 hour, from admission to 2 hours, and from 1 to 2 hours. Item-level outcomes comprised improvement, unchanged status, or worsening at 1 and 2 hours relative to baseline among patients with the corresponding baseline deficit.

Additional analyses quantified recovered and worsened NIHSS item-points from admission to 1 hour and from admission to 2 hours. Item-level change during the second hour, including improvement, unchanged status, worsening, and item-point changes among deficits persisting at 1 hour, was examined separately and is reported in the Supplementary Material. NIHSS at hospital discharge was reported as a later measure of neurological status and was not included in the fixed-time hyperacute repeated-measures analysis.

### Statistical analysis

Continuous and ordinal variables were summarized using medians and interquartile ranges (IQRs), with ranges reported where relevant. Categorical variables were summarized as counts and percentages.

Total NIHSS scores at admission, 1 hour, 2 hours, and discharge were summarized descriptively. Within-patient NIHSS changes from admission to 1 hour, admission to 2 hours, and 1 hour to 2 hours were summarized using the median, IQR, and range, together with the numbers and proportions of patients who improved, remained unchanged, or worsened.

Changes in total NIHSS across admission, 1 hour, and 2 hours were assessed using the Friedman test. Following a significant overall test, pairwise comparisons were performed using Wilcoxon signed-rank tests for admission versus 1 hour, admission versus 2 hours, and 1 hour versus 2 hours. To account for the three pairwise comparisons, a Bonferroni-adjusted significance threshold of *P* < 0.0167 was applied.

Item-level neurological evolution was summarized as counts and percentages using the predefined eligible denominator for each NIHSS component and interval. Recovered NIHSS item-points were summed by component, and the contribution of each component to total recovered item-points was expressed as a percentage. No formal inferential comparisons between individual NIHSS components were performed.

All statistical tests were two-sided. *P* < 0.05 was considered statistically significant, except for the Bonferroni-adjusted pairwise comparisons, for which *P* < 0.0167 was applied. Data management, preliminary calculations, and part of the statistical analyses were performed using Microsoft Excel. Further statistical analyses were conducted using jamovi version 2.7 (The jamovi project) [18].

## Results

### Study population

During the study period, patients with acute ischemic stroke involving the anterior circulation treated with IVT were screened. After application of the predefined inclusion and exclusion criteria, 124 patients were included in the analysis.

Median age was 67 years (IQR 59–77; range 32–94), and 62.1% were male. Median baseline NIHSS was 6 (IQR 5–9), and median ASPECTS was 10 (IQR 9–10). Median onset-to-needle time was 153.5 min (IQR 120–211.75). Median length of stay was 7 days (IQR 6–9; range 1–23). The prevalence of vascular risk factors and other baseline characteristics is presented in Table 1.

**Table 1:** Baseline characteristics of the study population

Characteristic	Value (*n* = 124)
Age (years), median (IQR); range	67 (59–77); range 32–94
Male sex, *n* (%)	77 (62.1)
Length of stay (days), median (IQR); range	7 (6–9); range 1–23
Left-sided stroke, *n* (%)	61 (49.2)
Right-sided stroke, *n* (%)	63 (50.8)
ASPECTS, median (IQR); range	10 (9–10); range 4–10
Pre-stroke mRS, median (IQR); range	0 (0–0); range 0–4
Onset-to-needle time (min), median (IQR); range	153.5 (120–211.75); range 26–278
Admission glucose (mg/dL), median (IQR); range	115 (100.75–142); range 76–312
Creatinine (mg/dL), median (IQR); range	0.925 (0.82–1.125); range 0.5–2.05
Dyslipidemia, *n* (%)	55 (44.4)
Peripheral artery disease, *n* (%)	2 (1.6)
Coronary heart disease, *n* (%)	17 (13.7)
Heart failure, *n* (%)	11 (8.9)
Chronic kidney disease, *n* (%)	6 (4.8)
Prior stroke, *n* (%)	17 (13.7)
Atrial fibrillation, *n* (%)	23 (18.5)
Type 2 diabetes mellitus, *n* (%)	21 (16.9)
Arterial hypertension, *n* (%)	98 (79.0)
Chronic gastroenterological disease, *n* (%)	18 (14.5)
Chronic respiratory disease, *n* (%)	15 (12.1)

Data are presented as median (IQR) with range, or as *n* (%). Total study population (*n* = 124). ASPECTS, Alberta Stroke Program Early CT Score; IQR, interquartile range; mRS, modified Rankin Scale; *n*, number of patients.

### Global NIHSS evolution

Median total NIHSS decreased from 6 (IQR 5–9) at admission to 4 (IQR 3–7) at 1 hour and 4 (IQR 2–5) at 2 hours. Median NIHSS at hospital discharge was 2 (IQR 1–4). At discharge, 20 of 124 patients (16.1%) had an NIHSS score of 0. The distribution of NIHSS scores is shown in Figure 1, and the corresponding median (IQR) values are presented in Table 2.

**Figure 1:**
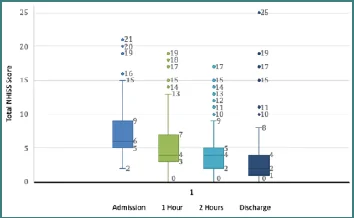
Median and interquartile range of total NIHSS scores across assessment time points

**Table 2:** NIHSS scores across assessment time points

Assessment time point	NIHSS, median (IQR)
Admission	6 (5–9)
1 hour	4 (3–7)
2 hours	4 (2–5)
Discharge	2 (1–4)

IQR, interquartile range; NIHSS, National Institutes of Health Stroke Scale.

From admission to 1 hour, median ΔNIHSS was −2 (IQR −3 to 0; range −12 to 7). Overall, 90 patients (72.6%) improved, 30 (24.2%) remained unchanged, and 4 (3.2%) worsened.

From admission to 2 hours, median ΔNIHSS was −2 (IQR −4 to −1; range −13 to 1). By 2 hours, 99 patients (79.8%) had improved, 21 (16.9%) remained unchanged, and 4 (3.2%) had worsened relative to baseline.

Between 1 and 2 hours, median ΔNIHSS was 0 (IQR −1 to 0; range −7 to 5). Nevertheless, 53 patients (42.7%) demonstrated further improvement, 66 (53.2%) remained unchanged, and 5 (4.0%) worsened during the second hour (Table 3).

**Table 3:** Total NIHSS evolution during the hyperacute period

Interval	ΔNIHSS, median (IQR)	Range	Improved, *n* (%)	Unchanged, *n* (%)	Worsened, *n* (%)
Admission → 1 h	−2 (−3 to 0)	−12 to 7	90 (72.6)	30 (24.2)	4 (3.2)
Admission → 2 h	−2 (−4 to −1)	−13 to 1	99 (79.8)	21 (16.9)	4 (3.2)
1 h → 2 h	0 (−1 to 0)	−7 to 5	53 (42.7)	66 (53.2)	5 (4.0)

Δ NIHSS was calculated as the follow-up NIHSS score minus the preceding NIHSS score; negative values therefore indicate improvement. Patients were classified as improved, unchanged, or worsened according to whether their total NIHSS score decreased, remained identical, or increased. h, hour(s); IQR, interquartile range; NIHSS, National Institutes of Health Stroke Scale.

Total NIHSS differed significantly across admission, 1 hour, and 2 hours (Friedman χ^2^(2) = 152; *P* < 0.001). Pairwise Wilcoxon signed-rank tests demonstrated significant differences between admission and 1 hour (*W* = 4290; *P* < 0.001), admission and 2 hours (*W* = 5302; *P* < 0.001), and 1 hour and 2 hours (*W* = 1534; *P* < 0.001). All three comparisons remained significant after Bonferroni correction. Although the median NIHSS was 4 at both 1 and 2 hours, the paired distribution continued to shift during the second hour, with 42.7% of patients demonstrating an additional reduction in NIHSS.

### Item-level hyperacute recovery

The frequency and temporal pattern of recovery varied across individual NIHSS components.

At 1 hour, the numerically highest improvement proportions were observed for LOC (6/10, 60.0%), LOC commands (13/22, 59.1%), and right-leg motor deficit (23/39, 59.0%). The lowest improvement proportions were observed for visual-field deficits (3/14, 21.4%) and facial palsy (22/96, 22.9%).

By 2 hours, improvement relative to baseline had occurred in 7/10 patients with impaired LOC (70.0%), 18/22 with impaired LOC commands (81.8%), 11/19 with gaze impairment (57.9%), and 5/14 with visual-field deficits (35.7%).

Among motor deficits, improvement at 2 hours was observed in 31/60 patients with left-arm weakness (51.7%), 28/49 with right-arm weakness (57.1%), 32/57 with left-leg weakness (56.1%), and 26/39 with right-leg weakness (66.7%).

Among higher-cortical deficits of interest, 19/44 patients with aphasia (43.2%) and 6/13 with extinction/inattention (46.2%) had improved relative to baseline by 2 hours.

For 14 of the 15 NIHSS components, the proportion classified as improved relative to baseline was numerically higher at 2 hours than at 1 hour. Limb ataxia showed the same improvement proportion at both assessments (28.6%). These between-item comparisons were descriptive. Complete item-level results are presented in Table 4.

**Table 4:** Item-level NIHSS evolution among patients with the corresponding baseline deficit

NIHSS component	Baseline deficit, *n*	At 1 hour			At 2 hours		
		Improved, *n* (%)	Unchanged, *n* (%)	Worsened, *n* (%)	Improved, *n* (%)	Unchanged, *n* (%)	Worsened, *n* (%)
1a. LOC	10	6 (60.0)	4 (40.0)	0	7 (70.0)	3 (30.0)	0
1b. LOC questions	42	22 (52.4)	19 (45.2)	1 (2.4)	25 (59.5)	17 (40.5)	0
1c. LOC commands	22	13 (59.1)	8 (36.4)	1 (4.5)	18 (81.8)	3 (13.6)	1 (4.5)
2. Best gaze	19	6 (31.6)	13 (68.4)	0	11 (57.9)	8 (42.1)	0
3. Visual fields	14	3 (21.4)	11 (78.6)	0	5 (35.7)	9 (64.3)	0
4. Facial palsy	96	22 (22.9)	74 (77.1)	0	34 (35.4)	62 (64.6)	0
5a. Motor arm, left	60	27 (45.0)	31 (51.7)	2 (3.3)	31 (51.7)	26 (43.3)	3 (5.0)
5b. Motor arm, right	49	23 (46.9)	26 (53.1)	0	28 (57.1)	21 (42.9)	0
6a. Motor leg, left	57	30 (52.6)	26 (45.6)	1 (1.8)	32 (56.1)	24 (42.1)	1 (1.8)
6b. Motor leg, right	39	23 (59.0)	16 (41.0)	0	26 (66.7)	13 (33.3)	0
7. Limb ataxia	7	2 (28.6)	5 (71.4)	0	2 (28.6)	5 (71.4)	0
8. Sensory	53	15 (28.3)	38 (71.7)	0	22 (41.5)	31 (58.5)	0
9. Best language	44	15 (34.1)	28 (63.6)	1 (2.3)	19 (43.2)	25 (56.8)	0
10. Dysarthria	59	20 (33.9)	39 (66.1)	0	24 (40.7)	35 (59.3)	0
11. Extinction and inattention	13	4 (30.8)	9 (69.2)	0	6 (46.2)	7 (53.8)	0

Percentages were calculated among patients with a non-zero score for the corresponding NIHSS component at admission; changes at 1 and 2 hours are relative to the baseline item score. LOC, level of consciousness; *n*, number of patients; NIHSS, National Institutes of Health Stroke Scale.

Among patients with a language deficit at admission, 44 had a non-zero NIHSS item 9 score. At 1 hour, the language score had improved in 15 patients (34.1%), remained unchanged in 28 (63.6%), and worsened in 1 (2.3%). By 2 hours, 19 patients (43.2%) showed improvement relative to baseline, and 25 (56.8%) remained unchanged, with no patient showing worsening relative to the admission language score.

### Contribution of individual NIHSS components to recovered item-points

Across baseline-positive deficits, 267 NIHSS item-points were recovered, and 6 item-points reflected worsening from admission to 1 hour, resulting in a net change of 261 points. From admission to 2 hours, 353 item-points were recovered, and 5 item-points reflected worsening, resulting in a net change of 348 points. During the first hour, the largest shares of recovered points were attributable to left-leg motor function (36 points; 13.5%), left-arm motor function (32; 12.0%), right-leg motor function (32; 12.0%), and LOC questions (27; 10.1%). By 2 hours, the largest shares were attributable to left-leg motor function (45; 12.7%), left-arm motor function (40; 11.3%), right-leg motor function (38; 10.8%), facial palsy (37; 10.5%), and right-arm motor function (37; 10.5%). Item-point changes are presented in Table 5.

**Table 5:** Recovered and worsened NIHSS item-points from admission to 1 and 2 hours among patients with the corresponding baseline deficit.

NIHSS component	Baseline deficit, *n*	Point sum	Admission to 1 hour			Admission to 2 hours		
			Recovered points	Worsened points	Net change	Recovered points	Worsened points	Net change
1a. LOC	10	12	6	0	6	7	0	7
1b. LOC questions	42	69	27	1	26	31	0	31
1c. LOC commands	22	30	15	1	14	20	1	19
2. Best gaze	19	28	8	0	8	14	0	14
3. Visual fields	14	20	5	0	5	8	0	8
4. Facial palsy	96	146	22	0	22	37	0	37
5a. Motor arm, left	60	110	32	2	30	40	3	37
5b. Motor arm, right	49	82	26	0	26	37	0	37
6a. Motor leg, left	57	106	36	1	35	45	1	44
6b. Motor leg, right	39	68	32	0	32	38	0	38
7. Limb ataxia	7	10	2	0	2	2	0	2
8. Sensory	53	57	15	0	15	23	0	23
9. Best language	44	79	17	1	16	21	0	21
10. Dysarthria	59	68	20	0	20	24	0	24
11. Extinction and inattention	13	15	4	0	4	6	0	6

Baseline (*n*) represents the number of patients with a score >0 for the respective NIHSS component at admission. Recovered points represent the summed magnitude of decreases in the corresponding item score, whereas worsened points represent the summed magnitude of increases. Net change was calculated as recovered points minus worsened points. LOC, level of consciousness; n, number of patients; NIHSS, National Institutes of Health Stroke Scale.

### Additional item-level recovery between 1 and 2 hours

Among patients whose corresponding baseline deficit remained present at 1 hour, further improvement during the second hour was observed across several NIHSS components. Numerically, the highest improvement proportions were seen for best gaze (7/15, 46.7%), LOC commands (5/14, 35.7%), and right-arm motor deficit (12/35, 34.3%). Among higher-cortical deficits, further improvement occurred in 5/42 patients with persistent language impairment (11.9%) and 2/9 with persistent extinction/inattention (22.2%). Overall, 104 NIHSS item-points were recovered during the 1-to-2-hour interval, while 7 item-points reflected worsening, resulting in a net change of 97 points. These findings were descriptive and based on the restricted second-hour denominator. Complete item-level results are provided in Supplementary Table S1.

Supplementary Material

## Discussion

In this cohort of 124 patients with anterior-circulation acute ischemic stroke treated with IVT, neurological improvement was already apparent during the first hour and continued over the first 2 hours. Median NIHSS decreased from 6 at admission to 4 at 1 hour and remained 4 at 2 hours. Improvement relative to baseline was observed in 72.6% of patients at 1 hour and in 79.8% at 2 hours. Importantly, the identical median NIHSS at 1 and 2 hours did not indicate neurological stability at the individual level. Between the 1- and 2-hour assessments, 42.7% of patients improved further, 53.2% remained unchanged, and 4.0% worsened. These findings show that neurological evolution may continue during the immediate post-thrombolysis period even when summary measures at the group level appear unchanged. The main contribution of the present analysis is therefore not to demonstrate that neurological status can change after thrombolysis, but to describe the extent and composition of that change across two routinely monitored early time points.

In the SITS-EAST registry, clinical recovery at 2 hours was defined as a reduction of at least 10 NIHSS points or a total NIHSS ≤3 and was observed in 31.0% of patients treated within 60 minutes of symptom onset, compared with 12.4% of those treated later [14]. In a cohort of 73 patients with anterior-circulation ischemic stroke treated with intravenous alteplase, Xiufu *et al*. reported a decrease in median NIHSS from 8 (IQR 5–12) before thrombolysis to 4 (IQR 3–8) at 1 hour. Twelve patients had an unchanged NIHSS at 1 hour, and none had a higher score than at baseline [19]. Akbulut *et al*. also evaluated neurological improvement at 1 hour, 24 hours, and 7 days after IVT, using a reduction of at least 4 NIHSS points to define improvement when analyzing factors associated with early improvement [20]. These studies differed in baseline severity, assessment schedule, and definition of response.

Early worsening has also been documented during the same phase. Seners *et al*. reported END of presumed ischemic origin within 24 hours in 12.1% of patients with minor stroke and large-vessel occlusion intended for IVT alone, and 48% of these deterioration events occurred within 2 hours after IVT initiation [5]. Shen *et al*. defined very early neurological deterioration as an increase of at least 4 NIHSS points during thrombolytic infusion and reported it in 8.4% of 345 treated patients [4]. These results cannot be directly compared with the 4.0% worsening observed between 1 and 2 hours in the present cohort because the populations, observation periods, and NIHSS thresholds differ. They do, however, provide a clinical frame for the second-hour changes observed here. In our analysis, any decrease or increase in total NIHSS was retained to describe the direction and extent of early neurological change, while item-level analysis characterized the neurological deficits underlying that change.

The continued evolution observed between 1 and 2 hours has practical relevance for post-thrombolysis monitoring. Serial neurological examination is already part of recommended post-IVT care, and the NIHSS provides a standardized means of documenting changes in neurological status [1]. The present findings do not support a new monitoring interval or intervention threshold, but they show that repeated structured examinations during this phase can capture both continued improvement and less frequent worsening. Worsening may arise through different mechanisms. In a prospective study of 168 IVT-treated patients, Nair *et al*. reported END, defined as an increase of ≥2 points in NIHSS at 24 hours, in 34 patients (20%), with ischemic deterioration occurring more frequently than hemorrhagic deterioration [21].

The vascular course after thrombolysis also provides context for early neurological change. Among 480 patients with proximal intracranial occlusion treated with intravenous alteplase, Tsivgoulis *et al*. reported early recanalization within 1 hour in 53%. Dramatic clinical recovery at 2 hours occurred in 54% of patients with early recanalization versus 10% of those without it [22]. Fu *et al*. found that extra-infarct and extra-penumbral growth at 24 hours were associated with unexplained END after reperfusion therapy, while absence of recanalization was associated with these adverse perfusion changes [23]. A systematic review by Mujanovic *et al*. reported cerebral no-reflow in 29% of patients considered to have successful macrovascular reperfusion, although definitions varied substantially across studies [24]. These mechanisms were not assessed in the present cohort and cannot be assigned to individual trajectories, but they provide a physiological context for the coexistence of improvement, persistent deficits, and worsening during the first hours after treatment.

The role of the 1- and 2-hour assessments should also be distinguished from the more established prognostic role of the 24-hour NIHSS. In the ENCHANTED study of 4410 thrombolysis-eligible patients, the absolute NIHSS at 24 hours had greater discriminatory ability for favorable 90-day functional recovery than baseline NIHSS and absolute or percentage NIHSS change [25]. The present 1- and 2-hour assessments address an earlier clinical phase. Their purpose is to characterize the hyperacute neurological trajectory during monitoring rather than to provide an alternative prognostic endpoint.

The additional information provided by the present study comes from examining the components underlying total-score change. Recovery was not uniform across NIHSS items. At 1 hour, the numerically highest improvement proportions were observed for LOC, LOC commands, and right-leg motor deficit, whereas visual-field impairment and facial palsy showed lower improvement proportions. By 2 hours, the proportion improved relative to baseline was numerically higher for 14 of the 15 NIHSS components. Among baseline deficits that remained present at 1 hour, further improvement during the second hour was most frequent for gaze, LOC commands, and right-arm motor function. These findings describe the neurological pattern underlying total NIHSS change and were not designed to establish differences in biological responsiveness between individual functions.

This item-level approach is consistent with literature emphasizing the information contained within individual neurological domains. Braun *et al*. highlighted differences in recovery and clinical consequences across stroke domains [6], while Hagberg *et al*. found that individual admission NIHSS items were associated with functional independence at 3 months and should be considered alongside the total score [7]. Cramer *et al*. noted that the total NIHSS may provide less insight into recovery within individual neurological domains than domain-specific endpoints [26]. Cheng *et al*. identified a five-factor NIHSS structure comprising left and right motor deficits, facial palsy/dysarthria, language-related deficits, and spatial attention/gaze deficits, which they described as a robust and generalizable representation of stroke deficit dimensions across populations, settings, and clinical phenotypes [27]. Several of these dimensions were represented among the baseline deficits in our cohort, further supporting item-level analysis alongside the total NIHSS. Ahmed *et al*. evaluated changes in language, motor deficits, and neglect among patients with large-vessel occlusion managed with thrombolysis, endovascular thrombectomy, combined therapy, or no reperfusion intervention [15]. In patients presenting with aphasia and/or right-sided weakness, language showed a greater proportional improvement than motor weakness (29.8% vs 12.7%; *P* < 0.0001), a pattern observed across treatment groups. Neglect was less frequent at baseline, but most affected patients also improved [15]. Although the population and treatment setting differ from the present IVT-only cohort, these findings further illustrate that recovery may vary across individual neurological functions. The present study extends this perspective to the complete NIHSS item profile in an IVT-only anterior-circulation cohort.

The functional consequences and management of individual neurological deficits differ across domains. Guidance from the National Institute for Health and Care Excellence recommends detailed assessment and appropriate speech and language intervention for post-stroke communication difficulties and physiotherapy for weakness, sensory disturbance, or balance problems affecting movement; it also emphasizes assessment of functional abilities and care needs after stroke [28].

Visuospatial neglect has been associated with reduced independence in activities of daily living, impaired self-care and mobility, reduced participation, and greater caregiver burden [9]. For post-stroke dysarthria, speech intelligibility, participation in conversation, living well with dysarthria, and communication-partner skills have been identified as core clinical outcome domains [29].

The recovered-item-point analysis addressed a related question: how much each NIHSS component contributed numerically to the observed score reduction. From admission to 1 hour, 267 NIHSS points were recovered and 6 reflected worsening, resulting in a net change of 261 points. From admission to 2 hours, 353 points were recovered and 5 reflected worsening, resulting in a net change of 348 points. Motor components contributed substantially to recovered points, but this should not be interpreted as preferential motor recovery. Point contribution depends on the prevalence and baseline severity of each deficit, the scoring range of the item, and the magnitude of subsequent change. Patient-level improvement proportions and item-point recovery therefore describe different aspects of the same neurological course. The large motor contribution in our cohort partly reflects the wider scoring ranges of these items. In contrast, several non-motor NIHSS items have lower maximum scores, such that even a one-point improvement may represent a clinically meaningful neurological change despite contributing fewer points to the overall recovered-point total (Table 5).

Language provides a clinically relevant example. Among 44 patients with a baseline language deficit, NIHSS item 9 improved in 34.1% at 1 hour and in 43.2% at 2 hours. Bueno-Guerra *et al*. reported poorer functional communication and quality of life among patients with post-stroke aphasia than among healthy controls [8]. However, NIHSS item 9 is not a comprehensive aphasia assessment. Grönberg *et al*. found a sensitivity of 72% for item 9 compared with dedicated language testing and reported that milder aphasia may be missed [11]. The RELEASE individual participant data meta-analysis showed that post-stroke language recovery can be characterized separately across overall language ability, auditory comprehension, naming, and functional communication, with recovery patterns differing according to age and time since stroke [30]. Improvement in NIHSS item 9 should therefore be interpreted as improvement in the language deficit captured by the NIHSS rather than complete recovery from aphasia. The same principle applies to other deficits. Visuospatial neglect affects activities of daily living and participation [9], while speech intelligibility and participation in conversation are among the core outcomes identified for dysarthria after stroke [29].

This distinction is particularly important for cognition. The NIHSS is not a cognitive screening instrument, and Abzhandadze *et al*. demonstrated limited sensitivity of an NIHSS-derived cognitive subscale for cognitive impairment [10]. Neurological status may nevertheless influence the feasibility of early cognitive assessment. In 881 patients assessed within 5 days of stroke, Suda *et al*. found that both the Mini-Mental State Examination and the Montreal Cognitive Assessment could be completed in 70.5% of patients and that a higher NIHSS score was independently associated with inability to complete testing [31]. These observations support considering the neurological state in the interpretation and feasibility of subsequent cognitive assessment, without treating the NIHSS as a measure of cognition.

Although laterality was not examined in the present study, future larger cohorts could assess whether hyperacute item-level trajectories differ by hemisphere and arterial territory, particularly given the reported left-hemisphere bias of the NIHSS in middle cerebral artery stroke [32].

Several features strengthen the descriptive value of the study, including predefined routine post-IVT assessments, restriction to an anterior-circulation IVT-only cohort, and parallel examination of total NIHSS change, patient-level item recovery, and item-point change. Several limitations must nevertheless be considered. This was a retrospective, single-center study with a moderate sample size, and some individual deficits were present in relatively few patients. Consequently, item-specific recovery percentages based on small denominators may be sensitive to changes in only one or two patients and should be interpreted cautiously.

The 1- and 2-hour assessments represented predefined routine monitoring categories rather than reconstructed measurements at exactly 60 and 120 minutes. No inferential comparisons were performed between individual NIHSS components, so numerical differences in item-specific recovery remain descriptive. The second-hour item analysis was conditional on a deficit being present at baseline and persisting at 1 hour. New abnormalities in items scored 0 at baseline were not represented in the item-specific recovery denominators, although their effects remained captured by total NIHSS change. Recovered and worsened item-points are also influenced by the scoring ranges of individual NIHSS components and should not be interpreted as equivalent across neurological domains.

Serial vascular and perfusion imaging was unavailable, preventing attribution of individual neurological trajectories to recanalization or tissue-perfusion changes. Finally, the cohort was selected by post-baseline criteria, introducing potential selection bias. Patients undergoing endovascular thrombectomy, those developing hemorrhagic transformation, and those who died during hospitalization were excluded, while an adequate discharge assessment was required. These criteria may preferentially retain patients with a more favorable early clinical course. The observed recovery and worsening rates should therefore be interpreted within the selected subgroup rather than as representative of an unselected IVT-treated population.

Overall, the present findings show that early reduction in total NIHSS is composed of heterogeneous changes across individual neurological deficits. Continued within-patient change between 1 and 2 hours supports the information provided by serial structured neurological assessment during routine post-thrombolysis care, while item-level analysis describes the composition of the total-score trajectory. Prospective studies combining serial neurological assessment with vascular and perfusion imaging, lesion topography, laterality, and standardized cognitive and functional follow-up are needed to determine the longer-term significance of these hyperacute recovery patterns and their association with subsequent care needs.

## Conclusion

In this selected cohort of patients with anterior-circulation acute ischemic stroke treated with IVT, early neurological evolution was heterogeneous at both the patient and item level. Although the group median NIHSS was identical at 1 and 2 hours, this summary measure masked continued within-patient change and differing trajectories across individual neurological deficits. Item-level analysis provided a more detailed neurological profile of hyperacute recovery, identifying which deficits improved, persisted, or worsened and how these changes contributed to the overall NIHSS trajectory. Beyond describing neurological status, this information may help contextualize subsequent monitoring, assistance, and rehabilitation needs.

The 1- and 2-hour assessments should be interpreted as measures of an evolving hyperacute neurological trajectory rather than as substitutes for the more established 24-hour NIHSS or dedicated domain-specific evaluation. The selection characteristics of the cohort should be considered when generalizing the findings to the broader IVT-treated stroke population. Future prospective studies are needed to determine the clinical and longer-term functional significance of these early recovery patterns.

## Data Availability

Further data are available from the corresponding author on reasonable request.
