# Supplementary material for: Hyperacute total and item-level NIHSS evolution after intravenous thrombolysis in anterior-circulation ischemic stroke: a Romanian tertiary stroke center cohort
Source: J Med Life. 2026 Sep;19(9):681–90. doi: 10.25122/jml-2026-0127 (PMC13746965; doi:10.25122/jml-2026-0127)
Supplement: Supplementary file 1 [file JMedLife-19-681-s001.pdf]

Table S1. Item-level NIHSS change from 1 to 2 hours among deficits persisting at 1 hour

NIHSS component	Persisting deficit at 1 hour, *n*	Patients, *n* (%)			Item-points			
		Improved	Unchanged	Worsened	Recovered	Worsened	Net change	Contribution to recovered points (%)
1a. LOC	6	1 (16.7)	5 (83.3)	0	1	0	1	0.96
1b. LOC questions	26	5 (19.2)	21 (80.8)	0	5	0	5	4.81
1c. LOC commands	14	5 (35.7)	9 (64.3)	0	5	0	5	4.81
2. Best gaze	15	7 (46.7)	7 (46.7)	1 (6.7)	7	1	6	6.73
3. Visual fields	11	2 (18.2)	9 (81.8)	0	3	0	3	2.88
4. Facial palsy	89	16 (18.0)	73 (82.0)	0	16	0	16	15.38
5a. Motor arm, left	53	11 (20.8)	39 (73.6)	3 (5.7)	14	4	10	13.46
5b. Motor arm, right	35	12 (34.3)	23 (65.7)	0	13	0	13	12.50
6a. Motor leg, left	45	11 (24.4)	33 (73.3)	1 (2.2)	15	2	13	14.42
6b. Motor leg, right	25	6 (24.0)	19 (76.0)	0	6	0	6	5.77
7. Limb ataxia	7	0	7 (100.0)	0	0	0	0	0
8. Sensory	40	7 (17.5)	33 (82.5)	0	8	0	8	7.69
9. Best language	42	5 (11.9)	37 (88.1)	0	5	0	5	4.81
10. Dysarthria	42	4 (9.5)	38 (90.5)	0	4	0	4	3.85
11. Extinction and inattention	9	2 (22.2)	7 (77.8)	0	2	0	2	1.92
Total	—	—	—	—	104	7	97	100

Note: The denominator included patients with the corresponding deficit at baseline who continued to have a non-zero score for that item at 1 hour. Patients whose baseline deficit had completely resolved by 1 hour were not included in the second-hour recovery denominator.

© 2026 by the authors. This article is an open access article distributed under the terms and conditions of the Creative Commons Attribution (CC BY 4.0) license.
